# Assessing removal force of abutment in dental implants: An *in vitro* study

**DOI:** 10.6026/973206300212590

**Published:** 2025-08-31

**Authors:** Amanda Antoinette Rebello, Sandeep Kumar, Mayur Kumar Soni, Bibhu Prasad Mishra, Neha Agrawal, Manvi Chandra Agarwal, Miral Mehta, Jugajyoti Pathi, Ramanpal Singh Makkad

**Affiliations:** 1 Department of Periodontology, Kalinga Institute of Dental Sciences, KIIT (Deemed to be University), KIIT Campus, Patia, Bhubaneswar, Odisha, India; 2 Department of Public Health Dentistry, Dental Institute, RIMS, Ranchi; 3 Department of Dentistry, Netaji Subhash Chandra Bose Medical College and Hospital, Jabalpur, Madhya Pradesh, India; 4 Department of Oral and Maxillofacial Surgery, Hi-Tech Dental College and Hospital, Bhubaneshwar, Odisha, India; 5 Department of Dentistry, Government Medical College, Mahasamund, Chhattisgarh, India; 6 Department of Periodontology and Implantology, Institute of Dental Sciences Bareilly, Uttar Pradesh, India; 7 Department of Pediatric and Preventive Dentistry, Karnavati School of Dentistry, Karnavati University, Gandhinagar, Gujarat, India; 8 Department of Oral & Maxillofacial Surgery, Kalinga Institute of Dental Sciences, KIIT Deemed to be University, Bhubaneswar, Odisha, India; 9 Department of Oral Medicine and Radiology, New Horizon Dental College and Research Institute, Bilaspur, Chhattisgarh, India

**Keywords:** Dental implant, abutment removal force, implant-abutment connection, *in vitro*, retention strength, universal testing machine

## Abstract

The removal force of abutments with various implant abutment connections is of interest. Hence, thirty implants were categorized into types, *i.e.*, external hex, internal hex and conical connections. The highest removal force was recorded in conical (192.7 +/- 10.9 N) and internal hex (174.3 +/- 15.4 N) and the lowest were in the external hex (145.8 +/- 12.6 N). Important disparities between groups were identified (p < 0.05). Thus, the conical connections provide better mechanical anchoring, thus improving the stability of the implants.

## Background:

The replacement of the missing teeth has become an acceptable and predictable method using dental implants. Osseointegration alone is not sufficient to assure the long-term success of implant-supported restorations, but anchorage between the implant and the abutment is also at stake [1]. One of the crucial elements that make dental implants successful is the use of the secure connection of the abutment to the implant body, which guarantees the functional transfer of the loads and limits the micro-movement that may result in the loosening of the screw or the extended loss of parts [2]. It is a factor that determines the mechanical behavior of the prosthetic components because of how the implant-to-abutment interface is designed. The normal kinds of connection are external hexagon, the internal hexagon, as well as the conical (more tapered) connections, all of which will have different benefits and mechanical features attached to them [3]. Traditionally, external hex connections are used, but they are susceptible to mechanical problems because of having minimal resistance to lateral forces. Internal connections, especially those that are conical, are more mechanically stable since they get to engage deeper and provide more friction and fit [4]. Repeated disconnection and reconnection of abutments as frequently necessitated in clinical practice (taking impressions or adjusting prosthesis) could impair the interface integrity and influence the removal force needed to remove the abutments [5]. This is especially applicable to the assessment of resistance to tensile, although it can affect the one-time functioning of the restoration when functioning loads are applied. Controlled assessment of the biomechanical functioning of the implant-abutment connection using standardized conditions is possible *in vitro*. The amount of removal force needed to unseat abutments may be used to establish an understanding of retentive strength and mechanical stability of alternative connection systems [6]. The design of the implant-abutment connection directly influences the magnitude of removal force required, with conical connections generally showing greater stability than internal or external hex designs [7]. Being aware of these forces, clinicians can make better choices regarding the abutment design, which would help them achieve better clinical outcomes and reduce complications, like screw loosening or the instability of the prosthetic [8]. The implant-abutment connections are exposed to a wide range of intraoral forces such as load in the axial direction, lateral loads and torsions, which may dislodge the connection with the lapse of time. It has been argued that the removal torque or removal force of an abutment is a dependable reflection of the retentive strength and mechanical stability of the implant-abutment complex [9]. It has been demonstrated that the preload generated when applying the torque is essential in opposing micro-movement and mechanical loosening induced, which, once damaged, could lead to peri-implant bone loss or failure of the prosthesis [10]. These stresses are dissipated at the implant-abutment interface with different designs of the connection. The conical (Morse taper) connection has a wedging effect plus a great surface contact and it produces such a frictional lock that cannot be loosened easily, unlike external hex types [11]. This mechanical interaction with the tight machining contributes to the decrease in micro gap and bacteria penetration at the interface, which increases the biological and mechanical success equally [12]. External hex, by contrast, utilizes the abutment screw more to oppose dislodging forces, thus possibly predisposing it to screw loosening during cyclic loading [13]. Repetitive insertion and removal of abutment screws as witnessed during clinical manipulations might change the surface contact or 'strip off the threads, leading to a physical difference in the pull-out force experienced later on during disengagements [14]. More to the point, the properties of the materials of the abutment and the implant, such as the elastic modulus in titanium and implant hardness of the surface the latter, also play an important role in the quality of the engagement and retention force [15]. Other studies also note that surface finishing and internal taper inclination affect the abutment mechanical interlocking and removal properties [16]. Therefore, it is of interest to develop the evidence-based recommendations as to clinicians to make possible the selection of abutments and the preference of connections to achieve durability and clinical predictions.

## Materials and Methods:

The present *in vitro* study aimed to determine the comparison and assessment of the removal force of abutments of three various dental implant connection systems, including external hex, internal hex and conical (Morse taper) connections. An altogether of 30 implant samples were utilized and randomly separated into three groups (n = 10 each group) and thus representing each connection type. Titanium dental implants were used, which have the dimensions of 4.0 mm diameter and 10.0 mm length and were commercially available. A custom jig was used to mount each of the implants in such a way that the positioning of each becomes standardized in self-curing acrylic resin blocks. Standard abutments made of titanium and paired with every individual implant type were attached and tightened to 30 Ncm with a calibrated torque wrench as recommended by the manufacturer. This was done in 10 minutes with the retightening of the abutments to take into consideration the initial settling effect. All the samples mounted were stored in the artificial saliva at 37 °C in the intraoral condition simulator (7 days). There was no mechanical or thermal cycling carried out to isolate the variable of the connection type on the removal force. The samples were allowed to incubate, after which each sample was mounted at the base of a universal testing machine (Instron, USA). It used a bespoke device to put the test to a vertical tensile load at a crosshead speed of 1 mm/min without deforming the abutment. The amount of force that was needed to fully dislodge the abutment from the implant was measured in Newtons (N). Peak load at the time of detachment was recorded as a removal force and a single test was done on each sample. Observed data were entered and measured through the SPSS software (version 25.0; IBM Corp., Armonk, NY, USA). Each group was calculated in terms of mean and standard deviation. The statistical differences between the three groups were achieved by one-way ANOVA and then the post-hoc Tukey test was used to carry out the pairwise comparisons. Any p-value below 0.05 was regarded as statistically significant.

## Results:

The mean removal forces (in Newtons) for the abutments connected to dental implants with external hex, internal hex and conical connections were recorded and compared. The results demonstrated a clear difference in the retention strength among the three types of implant-abutment interfaces. Table 1 (see PDF) shows the mean and standard deviation (SD) of removal forces for each connection group. The conical connection group exhibited the highest mean removal force (192.7 ± 10.9 N), followed by the internal hex group (174.3 ± 15.4 N) and the external hex group (145.8 ± 12.6 N). Table 2 (see PDF) presents the results of one-way ANOVA used to assess statistical significance among the groups. A statistically significant difference was observed in the removal force between the groups (p < 0.001). Table 3 (see PDF) shows the results of Tukey's post hoc test, which revealed significant pairwise differences between the external hex and internal hex groups (p = 0.004), external hex and conical groups (p < 0.001) and internal hex and conical groups (p = 0.032). Table 4 (see PDF) presents the frequency distribution of removal force ranges in each group. Most conical connection samples recorded removal forces above 190 N, while the external hex samples were predominantly in the 140-150 N range. These results suggest that conical abutment connections provide the highest resistance to removal, followed by internal hex and then external hex designs.

## Discussion:

This *in vitro* experiment was conducted to assess and compare the removal force of abutments with dental implants of three types of connections, namely external hex, internal hex and conical (Morse taper). The results manifested that the conical connection showed the greatest average removal force, which reflected better mechanical retention than the internal and external hex connections. These findings are not new, as there has been literature in the past that indicates that a conical connection promotes superior mechanical connection and stability because of the frictional fit between the implant and abutment [2]. Although externally hex connection has been in use throughout history, loosening of the abutment screw and micro-movements at the interface have been synonymous with their use [1]. The screw is the main stabilizing factor in the design and this might not withstand the functional loading stresses with time [4]. When compared, internal hex and conical designs transfer forces more uniformly and have increased locking; this decreases the possibility of loosening that can be experienced in components of cup designs [6]. This study also offers statistically significant differences that support the two mechanical advantages of the internal and conical designs. Interference connection, as in the conical (Morse taper) connection, has had good performance due to the cold welding effect of interference, due to the actual mechanical retention and minimal microleakage [8]. Such a close relationship not only enhances stability but also helps to reduce bacterial infiltration, which is another source of peri-implantitis and marginal bone loss [17]. Huddar *et al.* confirmed that conical connections exhibited the highest mean removal force, further supporting their superior mechanical stability compared to internal and external hex interfaces [18]. The effect of the repeated disengagement of abutments and reengagement used during the clinical procedures has been found to affect the screw preload negatively and the joint will be weaker in the long run [19]. It has been documented that repeated merging-de-merging operations of the screws may undermine screw stability and cause a decline in the removal force, particularly at external hex systems [20]. In comparison, the internal and conical systems have a higher resistance when compared to numerous cycles, as they are designed [21]. Removal forces also depend on the material properties. The use of titanium implants with well-machined and compatible abutments gives a better fit and a better mechanical behavior [22]. The current study eliminated the difference in material by utilizing standard titanium abutments and implants and thus, the difference in results was purely due to the design of the connection and not related to any differences in production. Findings on the study herein are also comparable with the findings by Kim *et al.* who proved that conical connections possess excellent micromechanical behavior when it comes to resisting micro-movement under cyclic loading [23]. On the same note, additional *in vitro* tests have established that Morse taper designs had a greater screw loosening threshold and superior load distribution under dynamic loading [24]. Another restriction of this study is that it is *in vitro* and so it does not fully imitate intraoral conditions that include thermal flux, occlusal effects and biological reactions. Such findings should be confirmed by future studies of mechanical cycling, thermocycling and clinical research, which are essential in the context of real-life situations.

## Conclusion:

The design of the implant and abutment connection has a substantive effect on the mechanical retention of the abutment. When retention and long-term mechanical stability are of special importance, clinicians might regard the option of using conical connections.
